# Antioxidant and Neuroprotective Effects of Paeonol against Oxidative Stress and Altered Carrier-Mediated Transport System on NSC-34 Cell Lines

**DOI:** 10.3390/antiox11071392

**Published:** 2022-07-18

**Authors:** Sana Latif, Seung-Hye Choi, Asmita Gyawali, Seung Jae Hyeon, Young-Sook Kang, Hoon Ryu

**Affiliations:** 1 Research Institute of Pharmaceutical Sciences and College of Pharmacy, Sookmyung Women’s University, Seoul 04310, Korea; its.sanairfan@gmail.com (S.L.); gyawaliashu35@gmail.com (A.G.); 2 Brain Science Institute, Korea Institute of Science and Technology, Seoul 02792, Korea; shchoi323@kist.re.kr (S.-H.C.); sjhyeon@kist.re.kr (S.J.H.)

**Keywords:** paeonol, neuroprotection, reduction-oxidation reactions, carrier-mediated transport system, organic anion transporter 1 (Oat1), plasma membrane monoamine transporter (Pmat), amyotrophic lateral sclerosis (ALS), NSC-34 cell lines

## Abstract

Paeonol is a naturally occurring phenolic agent that attenuates neurotoxicity in neurodegenerative diseases. We aimed to investigate the antioxidant and protective effects of paeonol and determine its transport mechanism in wild-type (WT; NSC-34/hSOD1^WT^) and mutant-type (MT; NSC-34/hSOD1^G93A^) motor neuron-like amyotrophic lateral sclerosis (ALS) cell lines. Cytotoxicity induced by glutamate, lipopolysaccharides, and H_2_O_2_ reduced viability of cell; however, the addition of paeonol improved cell viability against neurotoxicity. The [^3^H]paeonol uptake was increased in the presence of H_2_O_2_ in both cell lines. Paeonol recovered ALS model cell lines by reducing mitochondrial oxidative stress induced by glutamate. The transport of paeonol was time-, concentration-, and pH-dependent in both NSC-34 cell lines. Kinetic parameters showed two transport sites with altered affinity and capacity in the MT cell line compared to the WT cell line. [^3^H]Paeonol uptake increased in the MT cell line transfected with organic anion transporter1 (Oat1)/Slc22a6 small interfering RNA compared to that in the control. Plasma membrane monoamine transporter (Pmat) was also involved in the uptake of paeonol by ALS model cell lines. Overall, paeonol exhibits neuroprotective activity via a carrier-mediated transport system and may be a beneficial therapy for preventing motor neuronal damage under ALS-like conditions.

## 1. Introduction

Amyotrophic lateral sclerosis (ALS) is a fatal progressive neurodegenerative disease which results in damaging the motor neurons in the brain and spinal cord [1]. Damage to motor neurons leads to atrophy of muscle, diminished strength, and death of the affected patients. About 90% of ALS cases are spontaneous, while 10% are associated with mutations in genes of a dominant trait [2]. Prominently, the superoxide dismutase 1 (SOD1) Cu^+2^/Zn^+2^ gene mutation is specifically linked to ALS [3]. The pathophysiology of ALS involves the loss of motor neuron function, mitochondrial abnormalities in surviving neurons, aggregates of neurofilaments, and activation of glial cells [4,5,6]. Oxidative stress leads to prominent neuropathological changes in ALS patient’s spinal cord and animal models [1,2]. Superoxide anion causes oxidation of DNA, lipid, and protein. Two superoxide anions are catalyzed by SOD1, an antioxidant enzyme, and turn into hydrogen peroxide and oxygen. Because motor neurons are particularly prone to oxidative stress in ALS, prevention of oxidative stress by antioxidants may facilitate a pro-survival pathway and protect motor neurons [3,4,5]. Only two drugs, riluzole and edaravone, have been approved for the treatment of ALS by the United States Federal Drug Administration; however, there is an urgent need to discover new therapeutic agents that can help treat and alleviate the symptoms of ALS [7,8].

Paeonol, chemically known as 2-hydroxy-4-methoxyacetophenone, is a traditional Chinese medicine, and a phenolic component isolated from *Moutan Cortex* or root bark of genus *Paeonia suffruticosa Andrew*. Products derived from the natural sources possess unique diversity in their structure and chemical properties, therefore serving as novel therapeutic agent [9]. Paeonol exhibits various biological and pharmacological activities, including acting as an anti-inflammatory agent against neuroinflammation, colitis, and arthritis [10]. In addition to the treatment of diabetic encephalopathy, it serves as an anti-diabetic agent, and as an antiarrhythmic and immunity enhancer agent [11]. In diabetes, paeonol has been shown to inhibit the vascular endothelial growth factor [12] and therefore exhibits a potential role in the treatment of diabetic retinopathy [13]. Paeonol has shown promise in treating Alzheimer’s [14] and Parkinson’s diseases, depression, aging, and cerebral ischemic injury [15].

Paeonol has a neuroprotective function and plays an anti-inflammatory role by blocking the release of pro-inflammatory cytokines [16] and preventing the hippocampal death of neurons induced by lipopolysaccharides (LPS). In a study on N-methyl-D-aspartate receptors, paeonol was shown to provide protection to neurons of the rat hippocampus against injury induced by the withdrawal of oxygen and glucose [17]. Our previous research showed the altered transport of various amino acids, for instance, citrulline, lysine, tryptophan, and acidic drugs, including 4-phenylbutyric acid and valproic acid [18], as well as the energy-metabolizing compound carnitine in an ALS model cell line [19,20,21,22,23]. Since paeonol had a neuroprotective effect on activated microglial cells and hippocampal neurons, we assumed that studying the transport of paeonol in NSC-34 cell lines would bring new insights into its role in ALS. NSC-34 is a hybrid cell line (ALS model) with mutation in SOD1^G93A^ gene and has been used as an in vitro model for the study of mechanism of degeneration of motor neurons and potential drug therapy [24]. Neuroinflammation produced by reactive oxygen species (ROS), inflammatory cytokine dysregulation, and abnormalities in gene expression are all interrelated mechanisms of ALS pathology [25]. The maintenance of cellular homeostasis via oxidation–reduction reactions is important because oxidative stress damages cellular biomolecules, including nucleic acids and building blocks proteins [26]. Mitochondrial dysfunction is considered the prominent mechanism of motor neuron death in ALS, resulting in defective mitochondrial transport functions in the axons of motor neurons [27]. Previous research showed that ALS patients’ mitochondria have defective calcium homeostasis, resulting in the elevated production of ROS, which are ultimately associated with tyrosine nitration and alteration of carbonylated proteins [28].

Researchers have studied the chemical properties of paeonol showing a low molecular weight of 166.17 g/mol and melting point of 52 °C. Further, the pKa value of paeonol is 9.78 and log P is 1.98 [29]. Paeonol has poor bioavailability and solubility; thus, it cannot be easily transported across cell membranes and it is difficult to understand its transport mechanism in various disease conditions [29]. Previous studies on the uptake of paeonol in the blood–brain barrier (BBB) [30], inner blood–retinal barrier (iBRB), and conditionally immortalized rat retinal capillary endothelial cells (TR-iBRB) [13] have shown the expression of several carrier-mediated transporters, such as the organic cation/carnitine transporter (OCTN1-2) family, organic cationic transporter (OCT) family, and plasma membrane monoamine transporter (PMAT) family [30]. In addition to zwitterions, organic anion transporter (OAT) families also belong to solute carrier family 22 (SLC22). *OAT1/Oat1* mRNA was found to be considerably less abundant in rat, mouse, and human brains [31], but it has been discovered in the choroid plexus of mice [32].

The aim of the current study is to investigate the protective role of paeonol in ALS model cell lines against neurotoxicity induced by inflammatory cytokines and glutamate-induced mitochondrial oxidative stress and examine the transport characteristics and transporters involved in paeonol uptake in NSC-34 cells.

## 2. Materials and Methods

### 2.1. Radioisotope Compound and Chemical Reagents

The radiolabeled labeling agent [^3^H]paeonol (7.2 Ci/mmol) was procured from PerkinElmer (Boston, MA, USA). Other unlabeled reagent-grade chemicals, including paeonol, carnitine, cimetidine, 1-methyl-4-phenylpyridinium ion (MPP^+^), quinidine, verapamil, edaravone, clonidine, tramadol, acetyl-l-carnitine (ALC), tetraethyl ammonium (TEA), para-amminohippuric acid (PAH), and glutamic acid, were obtained from Sigma Aldrich (St. Louis, MO, USA). Lipopolysaccharide (LPS) was acquired from List Biological Laboratories (Campbell, CA, USA).

### 2.2. NSC-34 Cell Culture

ALS model cell lines were obtained from Prof. Hoon Ryu (KIST, Seoul, Korea) and classified into wild-type (WT, NSC-34/ hSOD1WT) cell line and mutant-type (MT, NSC-34/hSOD1G93A) cell line [33]. Motor neuron-like hybrid cell lines, known as NSC-34 cells, were transfected with the pCL-neo expression vector according to the previous study [34]. For the experiments, WT cells at passage #6–20 and MT cells at passage #5–19 were cultured and sub-cultured on collagen coated culture dishes type 1 and were grown in Dulbecco’s modified Eagle’s medium (Invitrogen, San Diego, CA, USA), which was supplemented with 10% fetal bovine serum (HyClone), 100 U/mL penicillin, and 100 µg/mL streptomycin (Invitrogen). An incubator with humidified atmosphere with 5% CO_2_/95% air and temperature 37 °C was used to incubate the cells according to the procedure, as described in the previous study [15].

### 2.3. Cell Viability Assay

To assess the survival rate of the cells after exposure to inflammatory cytokines and oxidative stressor, the MTT [3-(4, 5-dimethyldiazol-2-yl)-2, 5-diphenyltetrazoliumbromide] assay was conducted. ALS model cell lines were treated with glutamate (2 mM) and LPS (20 ng/mL) and H_2_O_2_ (300 µM), either alone or in combination with pretreatment of paeonol (100 µM) and incubated for 24 h. After the designated time, MTT (5 mg/mL) solution was added to each well, and the well plate was wrapped in the foil in the dark and incubated at 37 °C for 3 h. The formazan precipitation of MTT solution turned into the purple solution. The well plate was washed with phosphate-buffered saline (PBS) and dimethylsulfoxide was added. The absorbance was measured using an Infinite F200 PRO microplate reader (Tecan Trading AG, Männedorf, Switzerland) at a wavelength of 550 nM [23]. For the analysis of data, we compared the absorbance value in MT with the absorbance value in WT control treated with glutamate, H_2_O_2_, and LPS in the presence or absence of paeonol pretreatment.

### 2.4. Measurement of Mitochondrial Membrane Potential (ψ) and Oxidative Stress, and Immunocytochemistry

MitoTracker (Thermo Scientific, Waltham, MA, USA) staining (CMS-ROS) was performed to measure the mitochondrial membrane potential in the motor neuron cell line of ALS [35]. The cells were stained with MitoTracker-Red (0.1 μM) for 30 min prior to fixation and subjected to DAPI staining. The intensity of mitochondrial membrane potential was analyzed using NIH ImageJ software. MitoSox (Thermo Scientific, Waltham, MA, USA) staining was performed to measure the level of mitochondrial superoxide in the NSC-34 cell line. The cells were stained with MitoSox (2.5 μM) for 30 min prior to fixation and subjected to DAPI staining. The intensity of mitochondrial oxidative stress was analyzed using the NIH ImageJ software.

On the other hand, active caspase-3 (Cas-3) immunoreactivity was determined in the motor neuron cell line of ALS. Fixed cells were incubated for 30 min in a blocking solution (2% donkey/goat serum, 0.3% Triton-X100 in 0.1 M PBS) and were further incubated with anti-cleaved caspase-3 rabbit antibody (1:200; Cell Signaling, Danvers, MA, USA) at 4 °C for 24 h. After washing 3 times with PBS solution, the slides were incubated with goat anti-rebbit-488 (1:200; Abcam, Cambridge, MA, USA) at RT for 2 h. Photo-images were taken by using an Olympus epifluorescence microscopy (Olympus, Tokyo, Japan). The semi-quantitative analysis of immunoreactivity was performed by the ImageJ program.

### 2.5. In Vitro Uptake Study of [^3^H]paeonol in Motor Neuron like Cell Line

The [^3^H]paeonol uptake study was performed using a previously reported method [36]. Prior to the uptake, monolayer of confluent NSC-34 cells was washed thrice with extracellular fluid (ECF) buffer and adjusted to pH 7.4 and at 37 °C. Uptake was carried out by adding 200 µL of radiolabeled compound [^3^H]paeonol (347 nM) to each well with or without the addition of the inhibitor at 37 °C for 5 min [22]. Ice-chilled ECF buffer (4 °C) was used to terminate the uptake of isotope, and then the cells for solubilization were treated with 750 µL of 1 N sodium hydroxide plus PBS [19]. For protein assay, DC Protein assay kit (Bio-Rad, Hercules, CA, USA) was used. An aliquot was extracted from 1 N NaOH plus PBS using bovine serum albumin as a standard. A liquid scintillation counter (LSC) (LS6500; Beckman, Fullerton, CA, USA) was used for measuring the radioactivity of the compound by mixing 500 µL of the solution with 5 mL of Ultima gold [37].

### 2.6. Estimation of Kinetic Parameters of [^3^H]paeonol Uptake in ALS Model Cell Lines

For kinetic analysis of [^3^H]paeonol in NSC-34 cell lines, the Michaelis–Menten constant (K_m_) and the maximum uptake rate (V_max_) were estimated using Equation (1):

V = [V_max1_ · C/(K_m1_ + C) + V_max2_ · C/(K_m2_ + C)] + K_d_ · C, (1)

where V is the initial uptake rate of [^3^H]paeonol at 5 min and C is the concentration of unlabeled paeonol.

### 2.7. Small Interfering RNA (siRNA) Transfection Study in NSC-34 Cell Lines

In transection experiments with siRNA for Octn1/Slc22a4 and Octn2/Slc22a5, multidrug and toxic compound extrusion (MATE/Slc47a1), Pmat/Slc29a4, Oct2/Slc22a2, sodium-coupled monocarboxylate transporter 1, 2 (SMCT1/Slc5a8, and SMCT2/Slc5a12), Oat1/Slc22a6, and negative control (non-targeting pool) were transfected using Lipofectamine^®^ 2000 (Invitrogen) as a vehicle in ALS model cell lines for 24 h at a concertation of 200 nM. After the designated period, an uptake study was conducted according to a method described earlier [23].

### 2.8. Quantitative Reverse Transcription-Polymerase Chain Reaction

Isolation of RNA was performed using a Trizol-based RNA isolation kit (Hybrid-R) (Gene All, Seoul, Korea). cDNA was synthesized using the Superscript III First-Strand Synthesis System (Takara, Maebashi, Japan). Quantitative reverse transcription-polymerase chain reaction (qRT-PCR) analysis was done using a StepOnePlus Sequence Detection System (Bio-Rad) with SYBR Green PCR Core reagents (Bio-Rad). All experiments were performed in triplicate, each being repeated at least three times. The relative amount of target mRNAs was normalized to *GAPDH* levels in parallel. Information of the qPCR primers sequence is provided in Supplementary Table S1.

### 2.9. ALS Mouse and Immunohistochemistry

Male ALS transgenic mSOD1 (G93A) mice (the H1 high-expresser strain) were purchased (Jackson Laboratories, Bar Harbor, ME, USA) and bred with females with a similar background (B6/SJLF1). The experiments mainly used postmortem spinal cord tissue sections from WT and ALS mice. The animal study protocol of this study was approved by the institutional animal care and use committee (IACUC) of the Korea Institute of Science and Technology (approval no. KIST-2021-05-062).

To determine OAT1 (SLC22A6) immunoreactivity, 120–150 days old WT littermate (N = 5) and ALS transgenic mSOD1 (G93A) mice (n = 5) were perfused using 4% paraformaldehyde. Their spinal cords were processed for cryo-sectioning. Serial sections with 30 µm thickness of were performed using a cryostat. Lumbar spinal cord sections were selected for immunostaining to determine OAT1 level. First, the spinal cord sections were incubated in a blocking solution (2% donkey/goat serum, 0.3% Triton-X100 in 0.1 M PBS) for 1 h and were further incubated with OAT1 (SLC22A6) (1:200 dilution) (#ab135924) antibody in a blocking solution at 4 °C for overnight. After washing 3 times with PBS, the slides were processed for the amplification of immunoreactivity signal by a Vector ABC Kit (Vector Laboratories, Inc., Burlingame, CA, USA). The OAT1 (SLC22A6) signals were developed using 3, 3′-diaminobenzidine (DAB) chromogen (Thermo Fisher Scientific, Meridian Rockford, IL, USA). Images were taken by an epifluorescence optical microscope (Olympus, Tokyo, Japan). The semi-quantitative analysis of immunoreactivity was undergone using the NIH ImageJ program.

### 2.10. Statistical Analysis

Data were analyzed by the one-way ANOVA and Dunnett’s tests, as appropriate (post hoc test). Each data point is represented as the mean ± standard error of the mean (SEM). All statistical analyses were performed using Sigma Plot (12 version, Systat Software Inc., Richmond, CA, USA), and *p*-value < 0.05 indicates statistical significance.

## 3. Results

### 3.1. Cytotoxicity Induced by Pro-Inflammatory Cytokines and Neuroprotective Effect of Paeonol in ALS Model Cell Line

**Figure 1:**
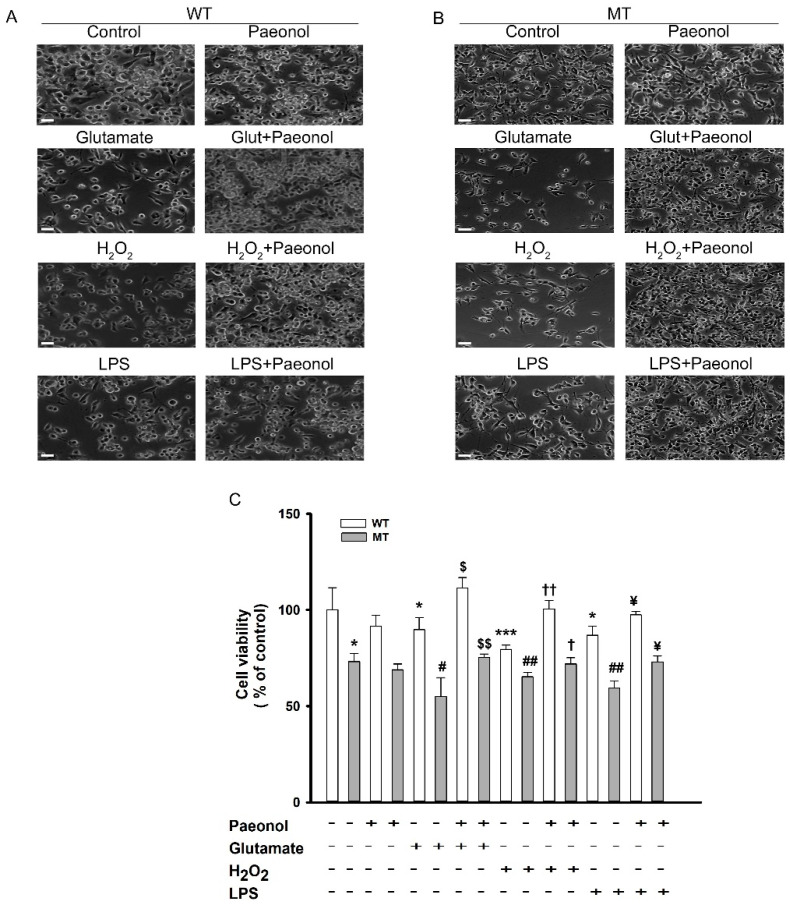
Effect of paeonol against the neuroinflammatory states in ALS model cell lines. Images of NSC-34 cell lines using EVOS XL Core Cell Imaging System (100×) in WT (**A**) and MT (**B**) cell lines. Cell viability using MTT assay in WT and MT (**C**) after 24 h treatment with glutamate (2 mM), H_2_O_2_ (300 µM), and LPS (20 ng/mL) with or without pretreatment with paeonol (100 µM). Each column represents the mean ± SEM (n = 3–4). * *p* < 0.05, and *** *p* < 0.001, significant different versus WT control; ^#^ *p* < 0.05 and ^##^ *p* <0.01, significant different versus MT; ^$^ *p* < 0.05 and ^$$^ *p* < 0.01, significant difference vs. glutamate; ^†^ *p* < 0.05 and ^††^ *p* < 0.01, significant difference vs. H_2_O_2_; ^¥^ *p* < 0.05, significant difference vs. LPS.

ALS pathophysiology exhibits that there is excess release of glutamate in the synaptic cleft, and astrocytes and microglia are unable to uptake the glutamate, resulting in excessive Ca^2+^ ion intake into the neuron [38]. There are several drugs that act on the various mechanisms to balance this release of glutamate. For instance, in ALS neurons, taurine shows the neuroprotective effect against glutamate-induced neurotoxicity [39]. Several other compounds, including riluzole, exhibit a protective role against glutamate-induced neurotoxicity [1]. Valproic acid also ameliorates the neurotoxicity induced by glutamate in NSC-34 motor neuron-like cells [4]. Together, to determine the neuroprotective effect of paeonol, we conducted glutamate- and inflammatory cytokine-induced cytotoxicity assay. ALS model cell lines were exposed to glutamate, inflammatory cytokines such as LPS, and the stress-inducing agent H_2_O_2_ for 24 h. Cell viability was markedly decreased in both cell lines; however, the co-addition of paeonol to glutamate, H_2_O_2_, and LPS significantly increased cell viability and restored it to normal levels in WT and MT cell lines (Figure 1A,B). The results indicate the neuroprotective effect of paeonol against glutamate and LPS neurotoxicity and its antioxidant effect against H_2_O_2_ in ALS model cell lines. The results from the MTT assay also showed similar results, and in both cell lines, treatment with only paeonol showed no substantial difference in the uptake of [^3^H]paeonol from the control (Figure 1C). This result indicates the non-toxic activity of paeonol in ALS model cell lines. Otherwise, post-treatment of paeonol was protective against H_2_O_2_-induced cell death (Supplementary Figure S1) but it was not protective against LPS or glutamate (data not shown).

**Figure 2:**
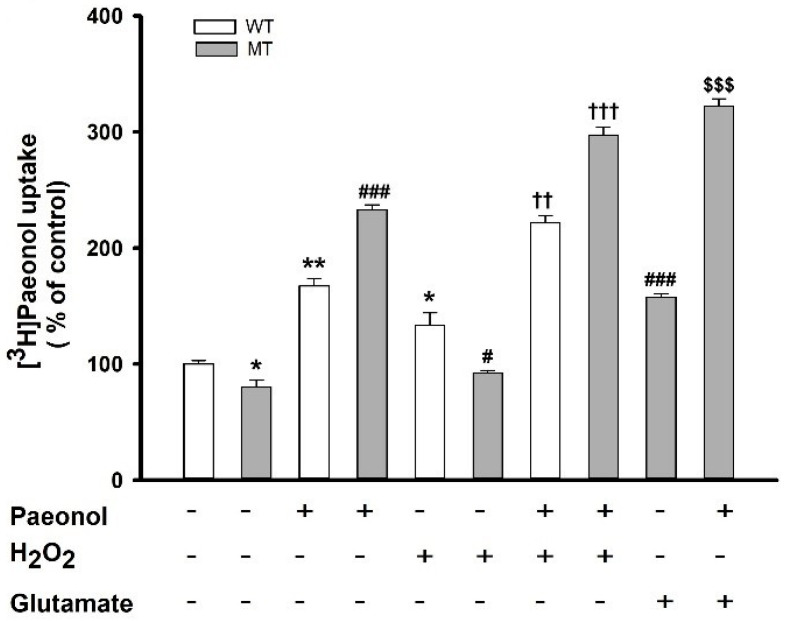
Treatment of H_2_O_2_ and glutamate and restoration effect of paeonol on the uptake of [^3^H]paeonol. [^3^H]Paeonol uptake in WT and MT cell lines pretreated with H_2_O_2_ (300 µM) either alone or in the presence of 100 µM of paeonol. MT cell line exposed to glutamate in the presence or absence of paeonol. [^3^H]Paeonol uptake was performed 24 h after pretreatment of cell lines at pH 7.4 and 37 °C. Each value represents the mean ± SEM (n = 3–4). * *p* < 0.05 and ** *p* < 0.01, significant difference vs. WT control; ^#^ *p* < 0.05 and ^###^ *p* < 0.001, significant difference versus MT (vehicle); ^††^ *p* < 0.01 and ^†††^ *p* < 0.001, significant difference vs. H_2_O_2_; ^$$$^ *p* < 0.001, significant difference vs. glutamate.

In our study, we assessed the uptake of paeonol after treating ALS model cell lines with H_2_O_2_. In both cell lines, the uptake of paeonol markedly increased; however, the addition of cold paeonol to these treated cells further increased the uptake of [^3^H]paeonol in both cell lines (Figure 2). Glutamate in the disease model cell line also showed a similar pattern.

### 3.2. Paeonol Preventing Glutamate-Induced Decrease of Mitochondria Membrane Potential (ψ) and Elevation of Mitochondrial Oxidative Stress in ALS Cell Line Model

**Figure 3:**
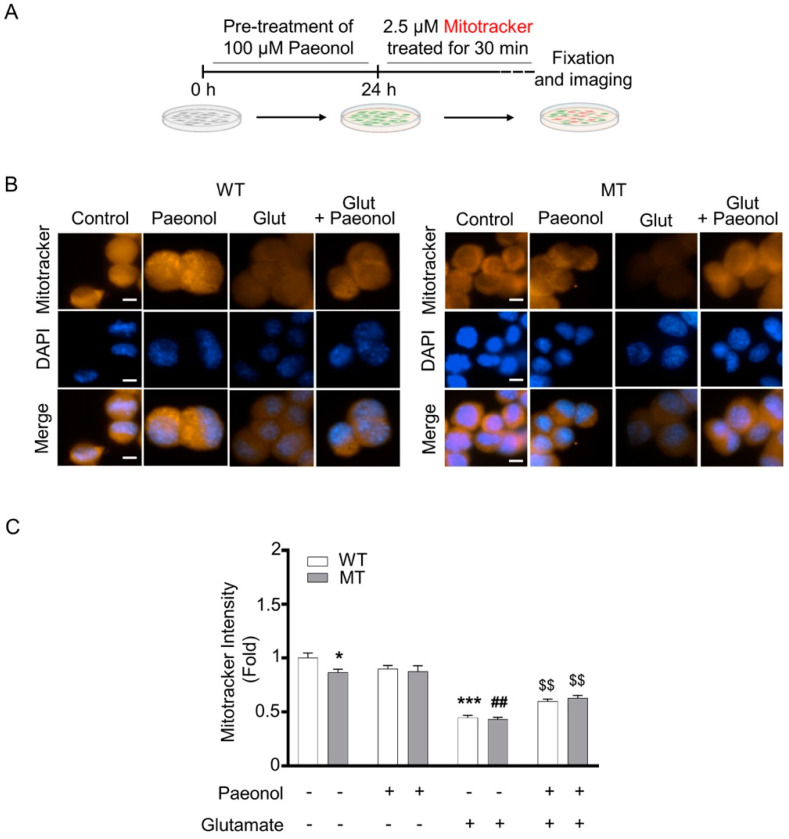
Paeonol prevents glutamate-induced decrease of mitochondrial membrane potential (ψ) in a cell line model of ALS. (**A**) A scheme presenting paeonol pre-treatment, glutamate treatment and MitoTracker staining. (**B**) Photo-images show that paeonol recovers glutamate-induced decrease of mitochondria membrane potential in WT and MT cell lines. Motor neurons were stained with MitoTracker-Red for 30 min prior to fixation and subjected to DAPI staining. (**C**) Paeonol significantly recovered glutamate-induced decrease of mitochondrial membrane potential. Cell count, n = 20 per each group. The graph data represent the mean ± SEM. * *p* < 0.05, and *** *p* < 0.001, significant different versus WT control; ^##^ *p* < 0.01, significant different versus MT (vehicle); ^$$^ *p* < 0.01, significant difference vs. glutamate. Scale bars (white): 10 μm.

**Figure 4:**
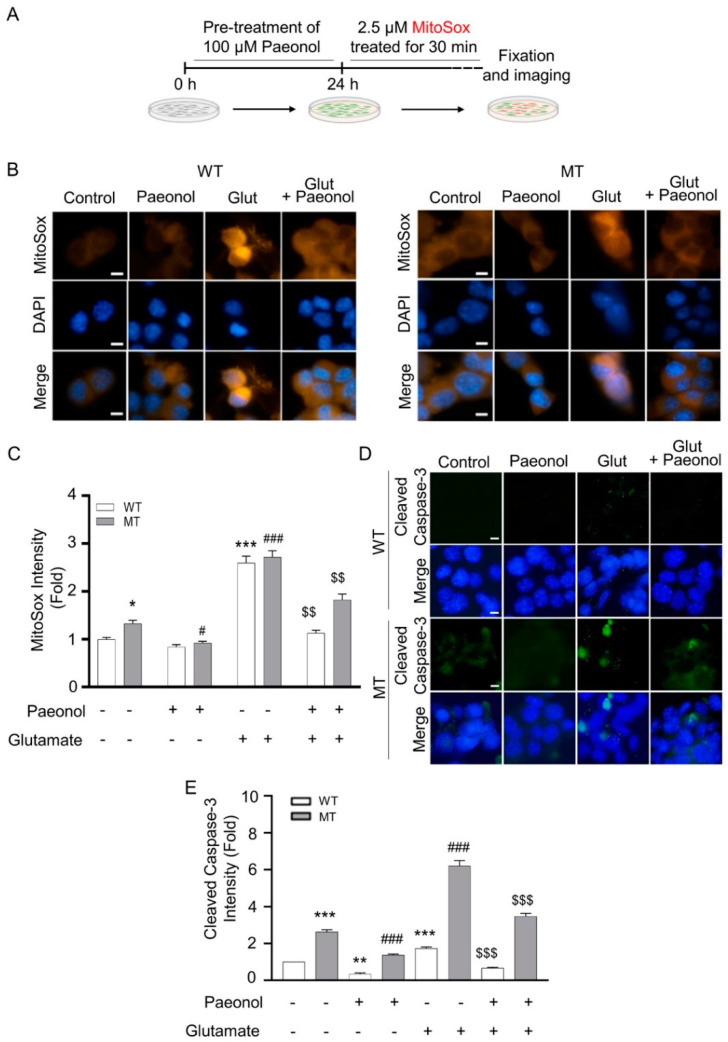
Paeonol prevents glutamate-induced elevation of mitochondrial oxidative stress in a cell line model of ALS. (**A**) A scheme presenting paeonol pre-treatment, glutamate treatment, and MitoSox staining. (**B**) Photo-images show that paeonol decreased glutamate-induced mitochondrial oxidative stress in WT and MT ALS cell lines. Motor neurons were stained with MitoSox for 30 min prior to fixation and subjected to DAPI staining. (**C**) Paeonol significantly reduced mitochondrial oxidative stress by glutamate. Cell count, n = 20 per each group. (**D**) Photo-images show that paeonol reduced glutamate-induced cleaved caspase-3, a cell death marker, in WT and MT ALS cell lines. (**E**) Paeonol significantly reduced cleaved caspase-3 by glutamate. Cell count, n = 30 per each group. The graph data represent the mean ± SEM. * *p* < 0.05, ** *p* < 0.01, and *** *p* < 0.001, significant difference at versus WT control; ^#^ *p* < 0.05, and ^###^ *p* < 0.001, versus MT (vehicle), ^$$^ *p* < 0.01 and ^$$$^ *p* < 0.001, significantly difference vs. glutamate. Scale bars (white): 10 μm.

Both motor neuron like cell lines were treated with paeonol for 24 h, and then motor neuron cell lines were treated with 0.1 µM MitoTracker staining for 30 min (Figure 3A). Figure 3 indicates that glutamate alone decreased mitochondrial membrane potential (ψ), whereas pretreatment with paeonol recovered the glutamate-induced loss in mitochondrial membrane potential in both cell lines (Figure 3B,C). ALS model cell lines were also pretreated for 24 h with paeonol and then with 2.5 µM MitoSox staining for 30 min (Figure 4A). MitoSox intensity was significantly increased in the presence of glutamate, whereas the addition of paeonol to glutamate resulted in a significant decrease in mitochondrial oxidative stress (Figure 4B,C). In order to determine whether paeonol can rescue motor neuronal damage against glutamate-induced cytotoxicity, we detected immunoreactivity of cleaved caspase-3, a well-known cell death marker, in WT and MT ALS cell lines. As expected, paeonol significantly reduced cleaved caspase-3 levels that were elevated by glutamate in both WT and MT ALS cell lines (Figure 4D,E).

### 3.3. Characteristics of [^3^H]paeonol Uptake by ALS Model Cell Lines

**Figure 5:**
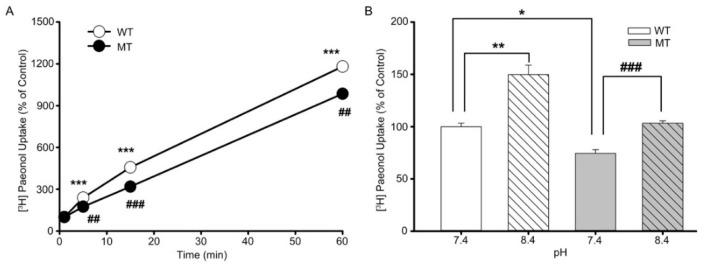
Characteristics of [^3^H]paeonol uptake are altered in NSC-34 cell lines. (**A**) [^3^H]Paeonol time course was observed for 0–60 min at pH 7.4 at 37 °C using ECF buffer solution in both WT and MT cell lines. (**B**) pH-dependent uptake was carried out for 5 min at pH 7.4 and 8.4 in both cell lines. Each value represents the mean ± SEM (n = 3–4). Significantly different at * *p* < 0.05, ** *p* < 0.01, *** *p* < 0.001. versus WT control; ^##^ *p* < 0.01, ^###^ *p* < 0.001, versus MT (vehicle).

Kinetic assessment was performed to characterize [^3^H]paeonol transport in NSC-34 cell lines. Initially, at pH 7.4 and temperature at 37 °C, the uptake of paeonol increased linearly until 5 min, showing time dependency in both the cell lines (Figure 5A). To assess the response of extracellular pH to paeonol uptake, pH was adjusted to physiological (7.4) and basic (8.4) levels. Figure 5B shows the uptake of paeonol divaricated with a change in pH from 7.4 to 8.4, and the uptake of paeonol increased with increasing pH in both cell lines, showing the pH dependency of paeonol in ALS model cell lines.

### 3.4. Analysis of Kinetic Parameters of [^3^H]paeonol Uptake by NSC-34 Cell Lines

**Figure 6:**
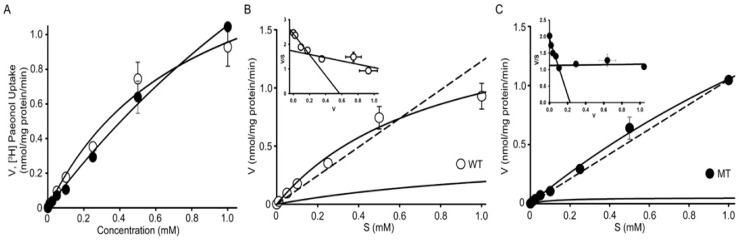
[^3^H]Paeonol concentration-dependent uptake in ALS model cell lines. (**A**) Uptake was carried out under physiological pH 7.4 and temperature 37 °C either in the absence or presence of cold paeonol at concentrations ranging from 0 to 1 mM for 5 min (**B**) in WT and (**C**) MT cell lines. Insets graphs are the Eadie–Hofstee plots (**B**,**C**). Each point represents the mean ± SEM (n = 3).

Kinetic parameters of paeonol in WT and MT cell lines were established using the Michaelis–Menten equation, and uptake was measured using the concentration of unlabeled paeonol ranging from 0 to 1 mM. The results revealed the increase in the uptake of paeonol with increasing concentration, thus showing concentration dependency of paeonol in ALS cell lines (Figure 6A). [^3^H]Paeonol uptake was lower in the MT cell line than that in the WT cell line. The estimated Michealis–Menton constants were 0.011 ± 0.009 and 0.098 ± 0.006 for high-affinity sites (K_m1_) and 0.821 ± 0.344 and 5.35 ± 1.52 (mM) for low-affinity sites (K_m2_) in WT and MT cell lines, respectively. Similarly, the maximum uptake rates (V_max1_) for high-affinity sites were 0.183 ± 0.019 and 0.161 ± 0.009 and for low-affinity sites (V_max2_) they were 1.82 ± 0.56 and 7.19 ± 1.50 (nmol/mg protein/min) in WT and MT cell lines, respectively (Table 1). These results suggest that at high-affinity sites, the MT cell line has higher affinity and lower capacity than the WT cell line, whereas at low-affinity sites, the MT cell line shows lower affinity and higher capacity. The Eadie–Scatchard inset graphs (Figure 6B,C) indicate that two carried mediated transport systems involved in the uptake of paeonol in ALS model cell lines. The results suggest that transport of paeonol is concentration-dependent and involves a carrier mediated transport system in both cell lines.

### 3.5. Effects of Pharmacological Drugs on the Paeonol Transport in ALS Model Cell Lines

To determine the effects of various compounds on the transport of paeonol in NSC-34 cell lines, we performed an uptake inhibition study using 1 mM of numerous drugs (Table 2). The uptake of [^3^H]paeonol was inhibited up to 65% by the cold paeonol; other compounds, including diphenhydramine, OCTN1 inhibitors such carnitine, cimetidine, and MPP^+^ showed significant inhibition in both cell lines. Quinidine, an antiarrhythmic Ca^+^ channel blocker and verapamil, known to be neuroprotective in ALS also showed marked inhibition. FDA-approved drug, edaravone which is used for the treatment of ALS, and clonidine, tramadol, and ALC also showed significant inhibition of [^3^H]paeonol uptake in both cell lines. However, TEA and PAH showed no significant inhibition in the WT control cell line, but the uptake of both TEA and PAH significantly increased in the MT cell line.

### 3.6. Knockdown by siRNA Transfection and mRNA Expression of Pmat, Octn1, and Oat1 in Cell and Animal Models of ALS

**Figure 7:**
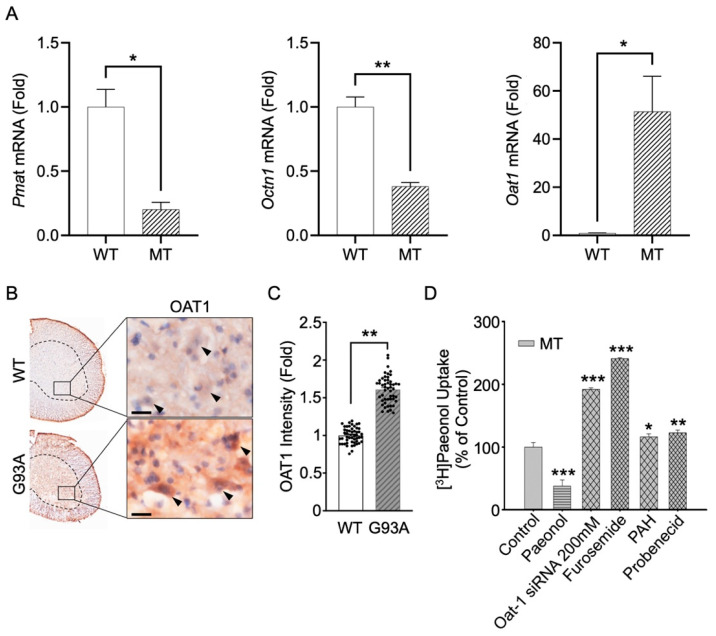
*Pmat* and *Octn1* genes are downregulated, whereas *Oat1* gene is upregulated in a cell line model of ALS. (**A**) qRT-PCR analysis confirmed that *Pmat* and *Octn1* mRNAs are significantly reduced, whereas *Oat1* mRNA is significantly increased in MT cell line compared to the control WT cell line. Target mRNA levels were normalized to *GAPDH*. The data represent the mean ± S.E.M of three separate experiments. * *p* < 0.05 and ** *p* < 0.01, significant difference vs. control (WT). (**B**) OAT1 immunoreactivity was decreased in the motor-neuron of G93A ALS transgenic mice compared to wild-type (WT). The nuclei were counterstained with hematoxylin. Scale bars (black): 20 μm. Arrowheads (black) indicate the OAT1 immunoreactivity in motor neurons. (**C**) The intensity of OAT1 was significantly increased in motor-neuron of G93A ALS transgenic mouse (each case, N = 5; cell counting, n = 10 (10 cells/case)). ** *p* < 0.01, significant difference vs. control (WT). (**D**) Effects of *Oat1* substrate/inhibitors on the uptake of paeonol. [^3^H]Paeonol uptake was carried out in the presence or absence of 1 mM paeonol (positive control) and 1 mM of furosemide, PAH, and probenecid. Oat1 siRNA transfection was carried out at 200 nM concentration in disease model (MT) cell line. Each point represents the mean ± SEM (n = 3–4). * *p* < 0.05, ** *p* < 0.01, and *** *p* < 0.001, significant difference vs. MT.

In order to investigate the involvement of transporters for the transport of paeonol in ALS model cell lines, we performed a siRNA transfection study. Gene knockdown will help in determining the specific transporter involved for the paeonol transport in ALS model cell lines. Various pools of targeted siRNAs were examined. Octn1, Octn2, SMCT1, and SMCT2 were expressed in NSC-34 cell lines and in the iBRB cell lines. Therefore, we chose to investigate siRNA transfection study Octn2, MATE, Oct2, SMCT1, and SMCT2, which showed no significant differences in the [^3^H]paeonol uptake in NSC-34 cell lines (Table 3). However, Octn1 significantly decreased the uptake of [^3^H]paeonol in MT cell lines. Pmat siRNA resulted in gene knockdown, with a significant decrease in the uptake of [^3^H]paeonol in both cell lines. Oat1, an antiporter transporter, showed a marked increase of up to 200% in the uptake of paeonol only in the disease cell line model of ALS, but did not affect the WT control cell line (Table 3). For the further confirmation of these results the mRNA expression level were performed, which revealed significant downregulation of *Pmat* genes in the MT cell line compared to that in the WT cell line. *Octn1* mRNA expression was considerably decreased in the disease model cell line compared to that in the control. The expression of *Oat1* mRNA was significantly higher in the MT cell line in comparison to the WT cell line (Figure 7A); therefore, *Oat1* genes were upregulated in the disease model cell line. In concurrence with the cell line model of ALS, the immunoreactivity of OAT1 in motor neurons of mSOD1(G93A) ALS mice was markedly increased (Figure 7B). The densitometry analysis showed that OAT1 intensity was significantly increase in motor neurons of mSOD1(G93A) ALS mouse spinal cord (Figure 7C).

These results prompted us to further analyze the uptake of Oat1 substrate and transporter inhibitors, including PAH as an Oat1 substrate, and probenecid and furosemide, which are Oat1 inhibitors (Figure 7D). Paeonol was taken as a positive control. The uptake of Oat inhibitors and substrates was increased significantly in the MT cell line, further confirming the outcome of the siRNA transfection and mRNA results. Hence, there might be some dysregulation or post-translational modification of the Oat1 transporter in the disease model cell line, and knockdown of Oat1 results in the efflux of the substrate in the cells, causing the increased [^3^H]paeonol uptake in the MT cell lines.

### 3.7. Determination of Minimal Inhibitory Concentration (IC_50_) of Drugs in Disease Model Cell Lines

**Figure 8:**
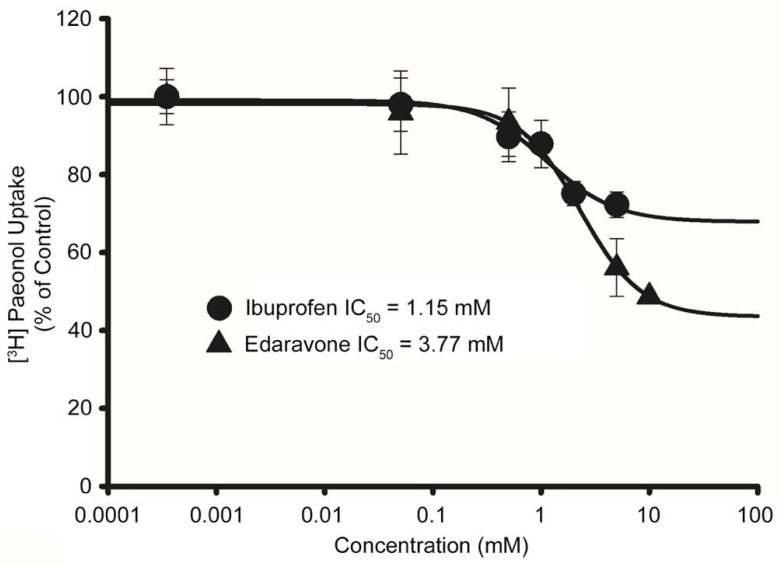
Effect of edaravone and ibuprofen in a dose–response manner (IC_50_) on the [^3^H]paeonol uptake in the ALS disease model cell line. The uptake was carried in the presence of edaravone and ibuprofen using varying concentration range 0–10 mM and at pH 7.4. All the data points are represented as mean ± SEM (n = 3).

We examined the interactions of drugs owing to their potential inhibitory effects on the [^3^H]paeonol uptake in disease model cell lines (Table 2). Therefore, to further evaluate the efficacy of the drugs, we determined the IC_50_ value of edaravone at 0.05–10 mM and ibuprofen at 0.05–5 mM in the MT cell line. Edaravone, an antioxidant and neuroprotective agent, exhibited inhibition in a concentration-dependent manner with the value of IC_50_ 3.77 mM, and ibuprofen, an anti-inflammatory drug, also showed concentration dependency inhibition with the value of IC_50_ 1.15 mM (Figure 8).

## 4. Discussion

Paeonol improves the condition of hippocampal neurons damaged by oxygen–glucose deprivation, plays a neuroprotective role in diabetic rats, and mitigates cognition deficits [40]. However, the effects of paeonol on ALS have not yet been studied, and we hypothesized that paeonol treatment may exert neuroprotective and antioxidant effects on NSC-34 cell lines against oxidative stress. In addition, we also investigated the transport characteristics of paeonol in ALS model cell lines.

### 4.1. Paeonol Restores Motor Neuronal Viability against Multiple Stresses

In the pathogenesis of ALS, neuro-inflammation plays an important role [2]. Therefore, in this study, we examined cell viability after treating cells with pro-inflammatory cytokine mediators, including glutamate, LPS, and H_2_O_2_. The results showed a remarkable decrease in the viability of cells in both cell lines treated with inflammatory cytokines. The addition of paeonol resulted in compensatory effects. Our current results are in line with the previous studies for determining neuroprotective effects of carnitine and taurine in NSC-34 cell lines [23,41]. L-carnitine, acetyl L-carnitine, and taurine supplementation prevents glutamate-induced neurotoxicity. It has been shown that paeonol exhibits neuroprotective effects against neurotoxicity induced by glutamate, owing to the inhibition of signaling pathways of apoptosis and p-ERK pathway upregulation in rat PC12 cells [42]. Paeonol also showed neuroprotective effects in diabetic encephalopathy by decreasing the caspase 3 expression and neuronal apoptosis in the cerebral cortex and hippocampus [40]. It has been studied previously that paeonol lessens the inflammation in microglia activated with LPS [43]. In addition, an earlier study has shown that paeonol exerts cellular protection by reducing the ROS production and improving the activity of SOD and upregulation of expression of Bcl-2 [44]. Research has shown that paeonol also repressed the death of cells induced by glutamate in rat pheochromocytoma (PC12) cells [42]. Pretreatment of paeonol in PIG1 cells showed the restored viability of the cell and reduced the oxidative stress by improving the SOD, glutathione peroxidase, and catalase [45].

### 4.2. Paeonol Modulates Mitochondria Function in ALS Cell Line

In ALS, oxidative stress has been considered as the major cause of death of neuronal cell due to the antioxidant enzyme SOD1 mutations, resulting in familial ALS [46]. Mitochondrial dysfunction have an important role in motor neurons [47]. The role of paeonol as an anti-inflammatory agent has been shown previously by reducing the production of ROS and slowing ATP-mediated raise in the migratory activity of cells. These actions of paeonol are linked to the regulation of glycogen synthase kinase 3 α/β and phosphorylated adenosine monophosphate-activated protein kinase-α [48]. In this study, we measured mitochondrial membrane potential using MitoTracker staining in motor neuron-like ALS model cell lines and showed that glutamate decrease the mitochondrial membrane potential in both cell lines. Glutamate also increased mitochondrial oxidative stress, as measured by MitoSox. Paeonol showed a compensatory effect by increasing mitochondrial membrane potential and decreasing glutamate-induced mitochondrial stress. Previous studies on cultured motor neurons from ALS transgenic animals suggest that the neurotoxicity induced by glutamate receptors is associated with reactive oxygen species production and mitochondrial Ca^+2^ overload [49]. Paeonol affects PC12 cells via lessening the mitochondrial glutamate-induced injury by normalizing the cytochrome c release and mitochondrial membrane potential [42].

### 4.3. Paeonol Uptake Is Differentially Regulated between WT and MT ALS Cell Line

In the uptake study, after pretreatment with the oxidizing agent H_2_O_2_, the uptake of [^3^H]paeonol increased in ALS model cell lines compared to that in the untreated cells, and the addition of cold paeonol resulted in a further increase in the uptake of paeonol in both cell line. Furthermore, glutamate significantly increased the uptake of paeonol in the MT cell line. In the NSC-34 cell lines, the paeonol transport was time-dependent and increased linearly with time up to 5 min. The uptake rate of [^3^H]paeonol was lower in the MT cell line in comparison to that in the WT cell lines. Our results showed the pH-dependent uptake of [^3^H]paeonol in NSC-34 cell lines. The uptake of paeonol significantly increased in an alkaline pH 8.4 as compared to the control pH of 7.4 in both cell lines. The results obtained are similar to the previous study of paeonol in TR-iBRB cell lines [23]. The concentration-dependent characteristics are regulated by a carrier-mediated transport system. The Eadie–Hofstee plot suggests the two saturable transport processes. The obtained results were similar to the previous study involving the PBA transport in motor neurons like cell lines [22]. The K_m_ and V_max_ values showed that the MT cell line at a high affinity site showed higher affinity and lower capacity compared to the WT cell line. The difference in the uptake of paeonol is caused by the relatively lower expression of transporters, mutation, or either deletion or resistance in carrier transporters, which occur in the disease model MT cell line rather than in the control WT cell line [23].

Next, we evaluated the effect of various drugs and substrates/inhibitors of transporters on the [^3^H]paeonol uptake. OCT/OCTN transporter function modulators, including MPP^+^, verapamil, and quinidine [50], inhibited the paeonol uptake significantly in both cell lines. In an earlier study, verapamil has shown to block the calcium ions and markedly lessen the aggregation of SOD1 [51]. MPP^+^ was mainly transported by dopamine transporters to dopaminergic neurons [52]. DPH, an antihistamine drug, was transported in a pH-dependent manner in Caco-2 cells [53]. In another study, diphenhydramine (DPH) was shown to inhibit serotonin uptake in human hepatocyte OCT-1 HEK293 cells [54]. In our study, significant uptake inhibition was observed in both cell lines. Similarly, the tertiary amine drug clonidine, which is transported by the H^+^ antiporter in the blood–brain barrier [55], markedly inhibited the transport of paeonol in NSC-34 cell lines. Additionally, OCTN transporter substrates, L-carnitine, and OCTN2 substrates [50] inhibited the uptake of paeonol in NSC-34 cell lines. Cimetidine, a substrate of P-glycoprotein [56], also inhibited [^3^H]paeonol uptake in both cell lines. Edaravone, a free radical scavenger, exerts neuroprotective effects by blocking lipid peroxidation [57]. In our study, 2 mM edaravone significantly inhibited both cell lines. TEA and PAH did not show any significant effects in the WT cell line. However, there was a significant increase in the paeonol uptake in the presence of TEA and PAH in the disease model MT cell line.

The altered transport characteristics of paeonol prompted us to further investigate the transporters responsible for the paeonol transport in ALS model cell lines. An earlier study on carnitine concluded the expression of *Octn1* and *Octn2* mRNA in ALS model cell lines [23]. Another research showed that OCTN1 has distinct characteristics as a cation/H ^+^ antiporter in the organic ion family of transporters [58]. Therefore, we studied the Octn1 siRNA transfection uptake of paeonol in an ALS cell line. Our results indicated significant change in paeonol transport in MT cell line. MATE, an cationic antiporter abundant at the membrane brush border, mediates tubular renal secretion of cationic drugs in collaboration with basolateral OCT2 [59]. In our study, siRNA transfection with both MATE and Oct2 showed no significant difference from the control siRNA, suggesting that both are not involved in the transport of paeonol in ALS model cell lines. Furthermore, we evaluated Pmat. It is profoundly expressed in the brain and transports xenobiotic cations and various biogenic amines [60]. In our study, Pmat showed a significant decrease in uptake in both cell lines. Therefore, we hypothesized that paeonol transport in ALS model cell lines is mediated by Pmat.

Next, we examined SMCT1 and SMCT2 siRNA transfection study, and the results indicated no marked effect on the paeonol uptake. The expression of SMCT1, which is a sodium-dependent transporter, has been previously found in NSC-34 cell lines [22]. Polyspecific transporter, Oat1, is required in the renal clearance of various endogenous compounds, toxins, and drugs [61]. The Oat1 siRNA knockdown study showed a marked increase in the uptake of paeonol only in the MT cell line, whereas no significant change in the transport was observed in the WT cell line. Previous studies have shown several reasons for the variations in Oats and organic anion transporting polypeptides (OATPs), including post-translational modifications, cellular trafficking, and other degradation mechanisms [62]. Therefore, we hypothesized that there might be some alteration or mutation in the diseased MT cell line that results in the elevated uptake of [^3^H]paeonol. Alterations in Oat and OATP functions or levels have been reported in liver disease and cancer [63]. These alterations might act as disease-modifying factors in patients and therefore can affect the pharmacokinetic profiles of drugs [63]. A previous study has reported that protein kinase C activation results in Oat1 retrieval by endocytosis from the plasma membrane [64]. Another study reported that protein kinase A activation caused an increase in the Oat1 mediated transport activity [65]. We compared our siRNA results with the mRNA expression of *Pmat*, *Octn1*, and *Oat1* in the ALS cell lines model, and the results showed downregulation of *Pmat* and *Octn1* and upregulation of the *Oat1* gene in the MT cell line. These findings are similar to our siRNA transfection analysis results, which revealed a significant decrease in the uptake of paeonol with Pmat siRNA transfection and Oat1 siRNA showed the marked elevation in the paeonol uptake in MT cell line. Furthermore, Octn1 mRNA expression was significantly lower in MT ALS cell line than in the WT cell lines. The siRNA transfection study showed significant change in the uptake of paeonol in MT ALS cell line.

We also examined the substrates and inhibitors of the Oat1 transporter to show the pattern they follow for the paeonol uptake in ALS model cell lines. PAH, a hydrophilic low-molecular-weight organic anion, significantly increased the uptake of [^3^H]paeonol. Previous kinetic analysis suggested that Oat1 contributes to the uptake of PAH in rat renal slices [66]. In addition, probenecid, an Oat1 inhibitor, has previously been investigated in rats and clinically in humans for renal excretion of drugs [67]. The current study showed that in the presence of probenecid there was a pronounced increase in the [^3^H]paeonol uptake in the MT cell line. Moreover, furosemide, a diuretic drug, has demonstrated a modulating effect on the expression of the Oat1 transporter [68] and considerably increased the uptake in the MT cell line as compared to the WT cell line. Earlier research has shown the *Oat1* and *Oat3* expression in the brain, and Oat family members, are reported as probenecid and PAH transporters. Oat3 facilitates the transport of indoxyl sulfate from the brain to the blood and plays a role in the efflux of drug metabolites and neurotransmitters [69]. Another study reported the involvement of the Oat family in the 17 β-estradiol-D-17 β-glucuronide efflux transport in BBB [70].

To improve the clinical applicability of drugs in the disease state, we evaluated the inhibitory concentrations of the drugs. Ibuprofen and edaravone concentrations of 1.15 and 3.77 mM, respectively, were required to inhibit by half [^3^H]paeonol uptake in the disease model cell line model. Ibuprofen, an anti-inflammatory drug, has an IC_50_ value of 1 mM in glioma cells [71], and edaravone, a novel antioxidant, reduces oxidative stress and acts as an antioxidant [72].

## 5. Conclusions

In the current study, we investigated the neuroprotective and antioxidant activities of paeonol against neurotoxicity induced by inflammatory cytokines in ALS cell lines. Paeonol pretreatment was found to reduce mitochondrial oxidative stress. The OCTN1, OAT1, and Pmat transporter showed altered behavior in the MT cell line. The transport characteristics of paeonol differed between the ALS cell lines. Our data indicate that Pmat may be responsible for the uptake of paeonol in the WT and MT ALS cell line. The findings imply that paeonol may serve as a potential neuroprotective and stress-reducing agent for the curative therapy of ALS.

## Figures and Tables

**Table 1:** Kinetics of [^3^H]paeonol uptake transporters in ALS model cell lines.

Parameters	WT	MT
K_m1_ (mM)	0.011 ± 0.009	0.098 ± 0.006
K_m2_ (mM)	0.821 ± 0.344	5.35 ± 1.52 *
V_max1_ (nmol/mg protein/min)	0.183 ± 0.019	0.161 ± 0.009
V_max2_ (nmol/mg protein/min)	1.82 ± 0.56	7.19 ± 1.50 **

K_m1_ and K_m2_ are the transport affinities, and V_max1_ and V_max2_ are the maximum transport velocities at high- and low-affinity sites, respectively. * *p* < 0.05, ** *p* < 0.01, represent significance levels of difference from the respective WT. Each value is represented as the mean ± SEM.

**Table 2:** Effects of various compounds on uptake of [^3^H]paeonol in ALS model cell lines.

Pharmacological Compounds	Conc. (mM)	[^3^H]Paeonol Uptake (% of Control)	
		WT (hSOD1^WT^)	MT (hSOD^G93A^)
Control		100 ± 3	100 ± 3
+Paeonol	1	36.8 ± 0.8 ***	35.4 ± 1.3 ***
+Edaravone	1	62.1 ± 7.0 ***	67.9 ± 5.4 ***
+Ibuprofen	1	--	78 ± 7 *
+L-Carnitine	1	82.7 ± 3.6 **	73.9 ± 2.3 ***
+MPP^+^	1	63.3 ± 6.3 ***	73.8 ± 2.7 **
+Quinidine	1	63.9 ± 8.4 *	73.4 ± 4.8 *
+Verapamil	1	75.2 ± 4.5 *	73.1 ± 7.5 **
+Diphenhydramine	1	77.5 ± 6.0 **	75.3 ± 5.3 **
+Clonidine	1	72.6 ± 6.0 *	83.7 ± 2.6 **
+Tramadol	1	82.9 ± 4.5 *	80.5 ± 6.7 ***
+Cimetidine	1	86.6 ± 2.3 *	87.3 ± 4.2 *
+ALC	1	73.4 ± 10.5 *	82.0 ± 6.4 **
+TEA	1	90.7 ± 1.8	146 ± 6.6 **
+PAH	1	102 ± 10	116 ± 5 *

[^3^H]Paeonol uptake in the absence (control) or presence of 1 mM inhibitor solutions was performed at 37 °C and pH 7.4 for 5 min. Each value represents the mean ± SEM (n = 3–4). * *p* < 0.05, ** *p* <0.01, and *** *p* < 0.001 indicate significant differences with respect to each control. MPP^+^, 1-methyl-4-phenylpyridiniumion; ALC, acetyl-L-carnitine; TEA, tetraethyl ammonium; PAH, para-aminohippuric acid.

**Table 3:** Effect of gene knockdown by siRNA transfection on the [^3^H]paeonol uptake in ALS model cell lines.

siRNA	[^3^H]Paeonol Uptake (% of Control)	
	WT	MT
Control	100 ± 4	100 ± 4
Octn1	105 ± 2	85.1± 10.4 ***^,###^
Octn2	104 ± 6	102 ± 3
MATE	94.9 ± 5.0	93.4 ± 4.8
Pmat	80.4 ± 8.5 *	77.5 ± 10.1 *
Oct2	102 ± 4	107 ± 5
SMCT1	100 ± 5	101 ± 1
SMCT2	104 ± 9	103 ± 6
Oat1	101 ± 2	193 ± 3 ***^,###^

ALS model cell lines transfected using lipofectamine with 200 nM control, Octn1, Octn2, MATE, Pmat, Oct2, SMCT1, SMCT2, and Oat1 siRNA’s for 24 h. [^3^H]Paeonol uptake was carried out at pH 7.4 for 5 min at 37 °C. * *p* < 0.05 and *** *p* < 0.001 denotes significant difference from each control, ^###^ *p* < 0.001 significant difference from WT. Each value obtained is represented as the mean ± SEM (n = 3–4).

## Data Availability

All data obtained from this study are included in the article.

## Supplementary Materials

The following are available online at https://www.mdpi.com/article/10.3390/antiox11071392/s1, Table S1: Sequence information of qPCR primers. Figure S1: Post-treatment of paeonol is protective against H_2_O_2_-induced cell death in motor neuron cell line (NSC-34).
