# Supplementary material for: Antioxidant and Neuroprotective Effects of Paeonol against Oxidative Stress and Altered Carrier-Mediated Transport System on NSC-34 Cell Lines
Source: Antioxidants (Basel). 2022 Jul 18;11(7):1392. doi: 10.3390/antiox11071392 (PMC9311606; doi:10.3390/antiox11071392)
Supplement: Supplementary file 1 [file antioxidants-11-01392-s001.zip › antioxidants-1779458-supplementary.pdf]

Supplementary Information

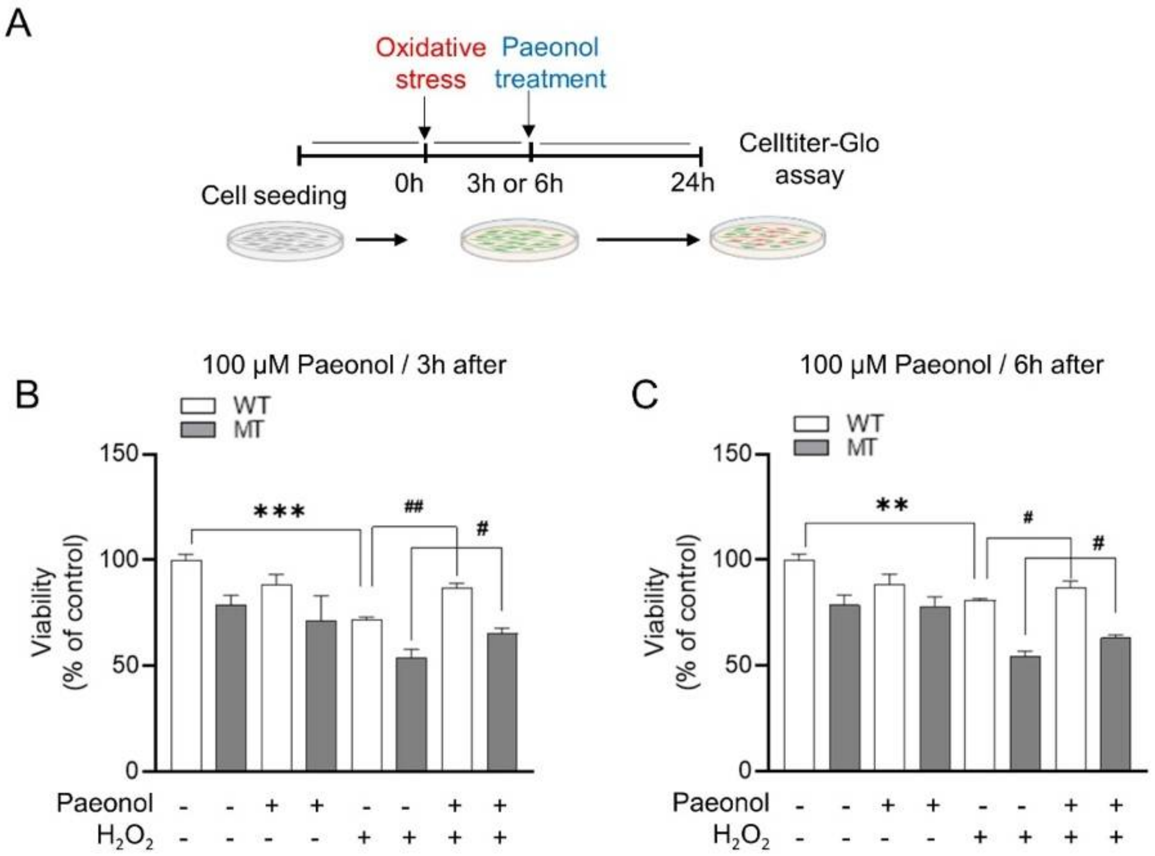

Supplementary Figure S1. Post-treatment of paeonol is protective against H_2O_2-induced cell death in motor neuron cell line (NSC-34). (A) A scheme illustrating the experimental flow of paeonol and H_2O_2 treatment *in vitro*. (B and C) Paeonol was treated in WT and MT cell line 3 h (B) after or 6 h (C) after treatment with H_2O_2 (300 μM). Cell viability was determined by CellTiter-Glo assay. Each graph represents the mean ± SEM of 3~4 separate experiments. Significantly different from control at $^{**}P < 0.01$, $^{***}P < 0.001$. Significantly different from H_2O_2 treated cells at $^{\#}P < 0.05$, $^{\#\#}P < 0.01$.
